# Combined image-guided radiofrequency and iodine-125 seeds implantation in the treatment of recurrent hepatocellular carcinoma after hepatectomy

**DOI:** 10.1186/s12885-024-12414-3

**Published:** 2024-05-31

**Authors:** Fei Cao, Jiaping Zheng, Weiyuan Hao

**Affiliations:** Zhejiang Cancer Hospital, Hangzhou Institute of Medicine (HIM), Chinese Academy of Sciences, Zhejiang Key Laboratory of Imaging and Interventional Medicine, Hangzhou, Zhejiang 310022 China

**Keywords:** Hepatocellular carcinoma, Recurrence, Radiofrequency ablation, Iodine-125 seeds

## Abstract

**Background:**

Currently, there is no consensus on the treatment of recurrent hepatocellular carcinoma (HCC) after hepatectomy. It is necessary to assess the efficacy and safety of radiofrequency ablation (RFA) combined with iodine-125 seeds implantation (RFA-^125^I) in the treatment of recurrent HCC.

**Methods:**

This study retrospectively analyzed the clinical data of patients with postoperative recurrence of HCC receiving RFA-^125^I or RFA treatment from January 2013 to January 2023. Both RFA and ^125^I seeds implantation were performed under dual guidance of ultrasound and CT. Overall survival (OS), progression-free survival (PFS), recurrence, and complications were compared between the two groups.

**Results:**

A total of 210 patients with recurrent HCC were enrolled in this study, including 125 patients in the RFA-^125^I group and 85 patients in the RFA group. The RFA-^125^I group showed a significantly better survival benefit than RFA group (median OS: 37 months vs. 16 months, *P* < 0.001; median PFS: 15 months vs. 10 months, *P* = 0.001). The uni- and multivariate analysis showed that RFA-^125^I was a protective factor for OS and PFS. There were no procedure-related deaths and no grade 3 or higher adverse events in both groups.

**Conclusions:**

RFA combined with ^125^I seeds implantation under dual guidance of ultrasound and CT is effective and safe for the treatment of HCC patients with recurrence after hepatectomy.

## Introduction

Hepatocellular carcinoma (HCC) poses a major global health challenge, being the sixth most prevalent cancer worldwide and the third leading cause of cancer-related death globally [1, 2]. Currently, the Barcelona Clinic Liver Cancer (BCLC) staging system is a widely accepted tool for prognostic prediction and treatment allocation for HCC [3]. Hepatectomy is a common and safe treatment option for patients diagnosed with early-stage HCC. Unfortunately, 50-70% of HCC patients have tumor recurrence within 5 years of hepatectomy, of which 61.4-83.3% have recurrence within 2 years. The 1-year and 5-year overall survival rates (OS) were 81.1% and 60.7%, respectively, for patients with recurrence, compared with 95.8% and 92.9%, respectively, for patients without recurrence [4, 5]. However, there is still no consensus on the treatment of recurrent HCC, and prescriptive treatment options are urgently needed, which is a thorny issue currently plaguing clinicians and patients.

For patients with recurrent HCC, re-resection or salvage liver transplantation remain the best treatment options. However, not all patients are suitable for surgical treatment because of the limited liver function reserve of the residual liver, postoperative adhesion, or lack of a liver donor [6, 7]. Radiofrequency ablation (RFA) has been accepted as an effective alternative to surgery in the management of small- to intermediate-sized (≤ 5 cm) HCC [8–10]. However, for the ablation of recurrent HCC in high-risk locations (tumors close to diaphragm, large vessel, liver capsule, gallbladder, gastrointestinal tract, or kidney), RFA seems to be difficult to achieve complete killing of tumors, which is often accompanied by tumor residual and easy to damage surrounding normal tissues, seriously affecting the prognosis of patients [11, 12]. Therefore, a more effective treatment strategy is needed to improve the efficacy of RFA for recurrent HCC.

Brachytherapy with iodine-125 (^125^I) seeds implantation for high dose irradiation of focal lesions has been widely used in the treatment of HCC and portal vein tumor thrombus [13, 14]. Studies have shown that ^125^I seeds can increase the efficacy of RFA in the treatment of HCC and is conducive to local tumor control [15]. However, as far as we know, there have been no reports on the treatment of recurrent HCC with RFA combined with ^125^I seeds (RFA-^125^I).

Lin et al. reported the use of MRI-guided RFA/^125^I seeds therapy for HCC, but the operation time was long and magnetic compatible puncture needles and RFA needles were required, which significantly limited its clinical application [16]. In addition, Chen et al. applied CT-guided microwave ablation (MWA) and ^125^I seeds implantation [17]. However, due to the inconsistent respiratory movements of patients under CT, the puncture angle and path need to be adjusted repeatedly. A real time and accurate imaging guidance is needed for RFA and iodine 125 particles implantation in the treatment of recurrent HCC. Therefore, the purpose of this study was to evaluate the efficacy and safety of RFA and ^125^I seeds implantation guided by ultrasound and CT in the treatment of recurrent HCC.

## Patients and methods

### Patients

The present study retrospectively analyzed the clinical data of 265 HCC patients who received RFA or RFA-^125^I at our center from January 2013 to January 2023. The patient’s treatment plan was recommended by the multidisciplinary Oncology Committee. For patients who refused ^125^I seeds implantation, treatment with RFA was performed. The present study was carried out in accordance with the principles of the Declaration of Helsinki. This retrospective study was approved by the institutional review board of the Zhejiang Cancer Hospital, Hangzhou Institute of Medicine (HIM), Chinese Academy of Sciences. Written informed consent was obtained from all patients prior to treatment.

Based on inclusion and exclusion criteria, 210 patients were eventually included in the study. Inclusion criteria were: (a) recurrent HCC patients older than 18 years of age; (b) a solitary HCC 3.0 cm in diameter or smaller or multiple (up to three) HCCs 3.0 cm in diameter or smaller; (c) The target lesion can be seen on ultrasound and CT, and the puncture path is safe; (d) Child-Pugh A or B; (e) there was no tumor vascular invasion, extrahepatic metastasis, refractory ascites or uncontrollable infection; (f) Eastern Cooperative Oncology Group (ECOG) 0 or 1; (g) patients were staged at BCLC-A in accordance with the BCLC system. Exclusion criteria were: (a) prior TACE, chemoradiotherapy, etc.; (b) accompanied by other malignancies; (c) perioperative clinical and imaging data were incomplete or lost to follow-up.

### RFA

**Fig. 1:**
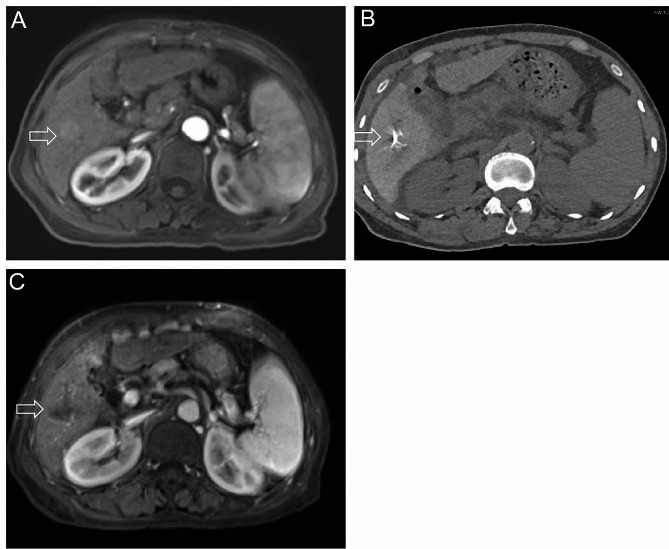
A 63-year-old male patient with HCC who underwent surgical resection six months ago has a recurrence of the tumor. (**A**) Enhanced MR showed a 1.8 × 2 cm tumor in the right lobe of liver. (**B**) The patient received RFA treatment; (**C**) MR reexamination 1 month later showed that the tumor was in complete response according to the modified Response Evaluation Criteria in Solid Tumors

^**125**^**I seeds implantation**.

In this study, the electrode needle was inserted into the target lesion under ultrasound and CT guidance (Fig. 1). Then turn on the RITA 1500 generator (RITA Medical Systems Inc., Mountain View, USA) and start the ablation. Select a single extendable electrode (less than or equal to 2 cm) or a multi-hook probe (greater than 2 cm) according to the tumor size. In order to achieve a safe range of 0.5–1.0 cm, multiple overlapping ablation zones are sometimes necessary. The analgesia was conducted by local injection of 5 mL of 2% lidocaine and intravenous administration of 50–100 mg of a flurbiprofen axetil injection (Tide Pharmaceutical Co., Ltd., Beijing, China).

**Fig. 2:**
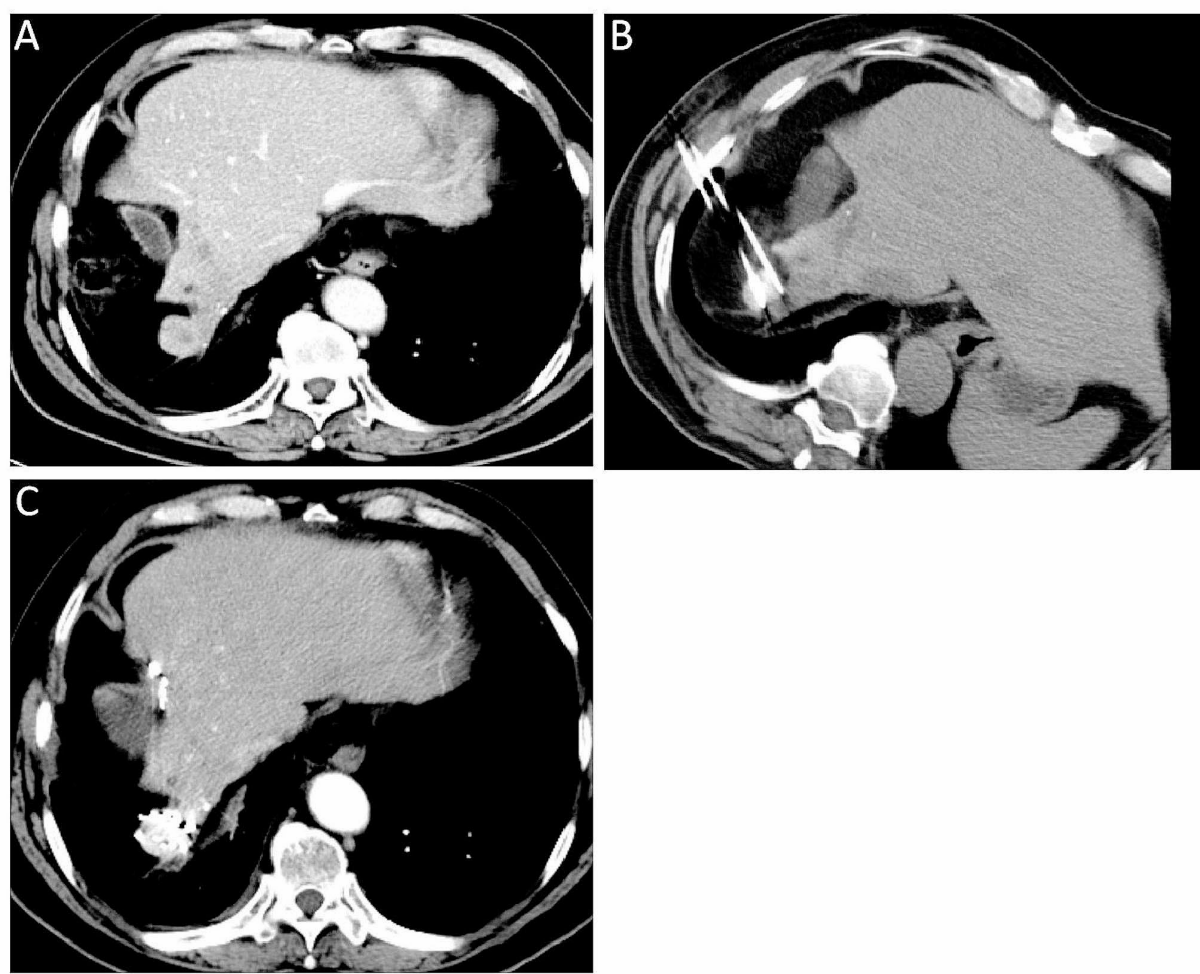
A 58-year-old male patient with HCC was diagnosed with recurrence 3 months after surgical resection. (**A**) Enhanced CT showed a 1.5 × 1.3 cm tumor in the right lobe of liver. (**B**) Immediately after RFA, the patient received I^125^ seeds implantation therapy; (**C**) CT reexamination 1 month later showed complete response according to the modified Response Evaluation Criteria in Solid Tumors

CT scans were performed immediately after RFA to assess the extent of ablation and possible residual tumor areas. The puncture needle was inserted into the target area under the dual guidance of CT and ultrasound (Fig. 2). In this study, Treatment Planning System (TPS; HGGR300, Hokai Medical Instruments Co., Ltd., Zhuhai, China) was used to determine the number and total activity of ^125^I seeds implanted. X-rays and γ-rays can reach the intended target volume, including tumors and 0.5–1.0 cm of the adjacent normal tissue. After ^125^I seeds implantation, CT scans were performed again to assess ^125^I seeds position and the presence of complications, while TPS was used for dose verification.

### Follow-up

Abdominal contrast-enhanced CT/MR and blood tests such as liver and kidney function, blood routine, and tumor markers were performed 4–6 weeks after the initial RFA. CT/MR evidence of recurrent or residual tumors included enhanced images of the arterial or portal venous phase of the tumor, and then repeated RFA or ^125^I seeds were applied. If there is no residual tumor, then the patient will be re-examined about 3 months, and the follow-up of this study was ended in January 2024.

The present study evaluated local and intrahepatic tumor recurrence. Local recurrence was defined as the appearance of tumor staining at the edge of the target lesion on CT/MR images of follow-up, and intrahepatic recurrence was defined as the appearance of a single new lesion in the liver more than 2.0 cm away from the target lesion on these images. Progression-free survival (PFS) was defined as the time from initial treatment to tumor progression, patient death, or end of follow-up. OS was the time from initial treatment to death or the end of follow-up. The adverse events related to the treatments were assessed according to the Common Terminology Criteria for Adverse Events Version 5.0.

### Statistics analyses

Mean ± standard deviation was used to represent the continuous data, and Student’s *t test* was used to compare the difference between the two groups of continuous data. The percentage was used to represent the categorical variables, and the Chi-square test was used to compare the differences between the two groups of categorical variables. Cumulative OS and PFS were estimated by the Kaplan-Meier method and compared by log-rank test. multivariate Cox proportional hazards regression analysis was applied to evaluate prognostic factors affecting OS and PFS. The statistical significance was two-tailed, and a *P* value less than 0.05 was considered statistically significant.

## Results

### Study population

**Fig. 3:**
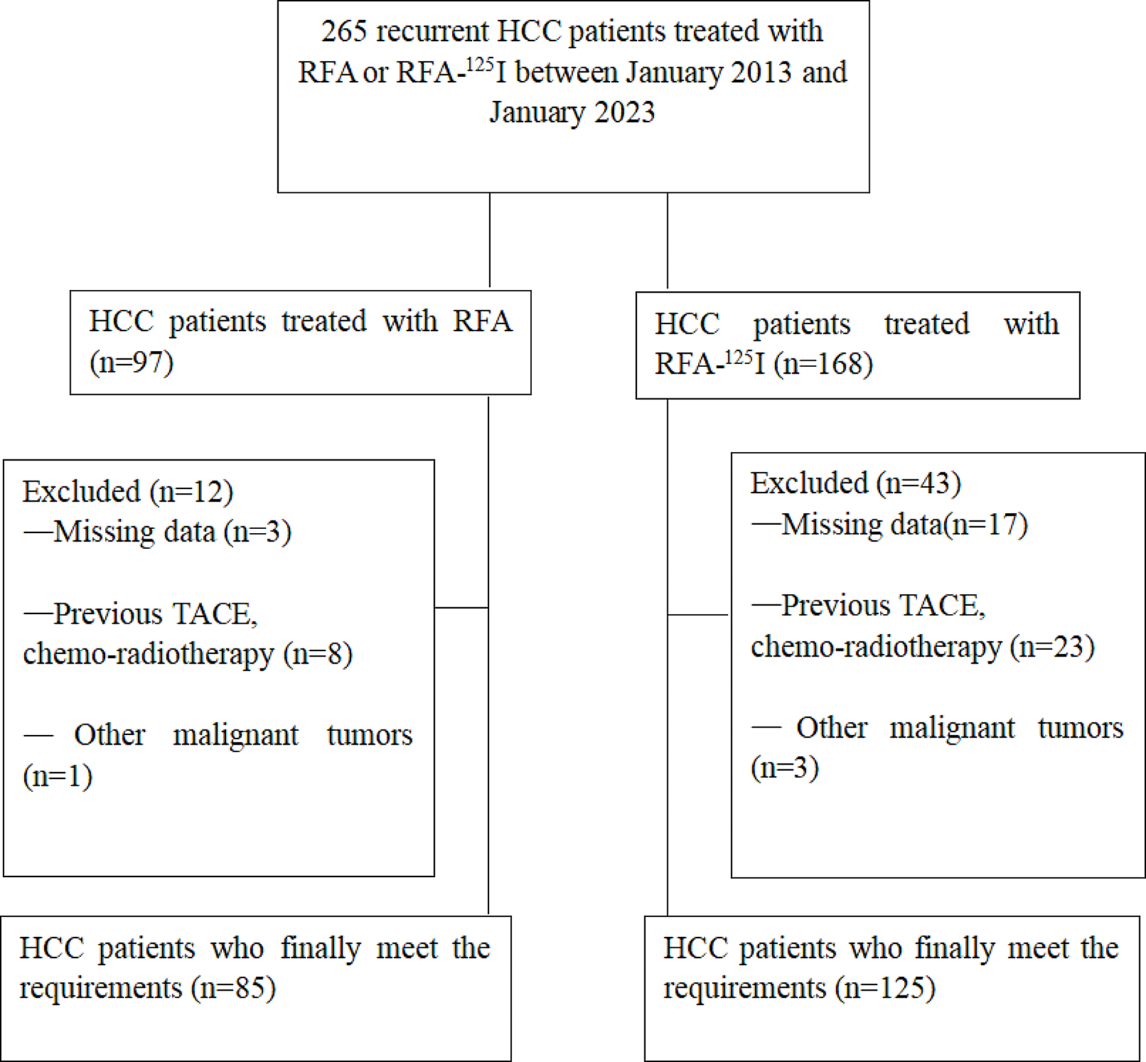
Flow chart shows the screening procedure for recurrent HCC patients

From January 2013 to June 2023, a total of 210 HCC patients were enrolled in this study, including 85 patients in the RFA group and 125 patients in the RFA-^125^I group (Fig. 3). In the RFA-^125^I group, a total of 2523 seeds were implanted, an average of 20.2 ± 9.5 per patient. The detailed baseline characteristics of the two groups of patients are shown in Table 1.

**Table 1:** Baseline characteristics

Characteristics	RFA-^125^I group (*N* = 125) (No, %; Mean ± SD)	RFA group (*N* = 85) (No, %; Mean ± SD)	*P* value
Gender			0.233
Male	109 (87.2%)	69 (81.2%)	
Female	16 (12.8%)	16 (18.8%)	
Age (years)	55.8 ± 10.7	55.6 ± 10.9	0.914
Hepatitis			0.540
Hepatitis B	105 (84.0%)	74 (87.1%)	
Other	20 (16.0%)	11 (12.9%)	
Child-Pugh score			0.448
A	96 (76.8%)	69 (81.2%)	
B	29 (23.2%)	16 (18.8%)	
TB (µmol/L)	21.5 ± 14.0	18.2 ± 10.0	0.065
Albumin (g/L)	37.5 ± 5.9	36.6 ± 5.1	0.255
PT(s)	14.0 ± 1.6	14.1 ± 1.5	0.758
AST (µmol/L)	42.1 ± 34.8	47.1 ± 35.4	0.314
ALT (µmol/L)	42.1 ± 34.8	47.1 ± 35.4	0.314
PLR	129.6 ± 105.3	135.7 ± 74.6	0.646
NLR	3.3 ± 3.4	3.2 ± 2.4	0.748
Tumor size (cm)	2.4 ± 0.7	2.2 ± 0.6	0.086
Tumor number			0.725
1	101 (80.8%)	67 (78.8%)	
2–3	24 (19.2%)	18 (21.2%)	
α-Fetoprotein level			0.456
>400 ng/mL	55 (44.0%)	33 (38.8%)	
≤ 400 ng/ml	70 (56.0%)	52 (61.2%)	

Note. RFA: Radiofrequency ablation; SD: Standard deviation; BCLC: Barcelona Clinical Liver Cancer; TB: Total bilirubin; PT: Prothrombin time; AST: Aspartate aminotransferase; ALT: Alanine aminotransferase; PLR: Platelet-to-lymphocyte ratio; NLR: Neutrophil-to-lymphocyte ratio

The study was followed up until January 30, 2024, with a median follow-up time of 13 months (range, 2–61 months) in the RFA group and 31 months (range, 4–84 months) in the RFA-^125^I group, respectively. During follow-up, 61 and 88 patients died in the two groups, respectively.

### Complications

There were no procedure-related deaths and no grade 3 or higher adverse events in both groups. All these symptoms were grade 1,2, and were significantly improved or disappeared after symptomatic treatment. Pneumothorax occurred after puncture in 4 patients, including 3 patients in the RFA-^125^I group, but all of them were grade 2. The patients had no discomfort such as dyspnea and were improved after conservative treatment. No migration of seeds from the liver to other organs was observed during follow-up.

### Recurrence

During follow-up, a total of 163 patients had recurrences, including 72 patients in the RFA group and 91 patients in the RFA-^125^I group. In the RFA group, 20 patients had local recurrence, 35 patients had intrahepatic recurrence, and 17 patients had extrahepatic metastasis, compared with 10, 37, 44 patients in the RFA-^125^I group, respectively.

### Overall survival

**Fig. 4:**
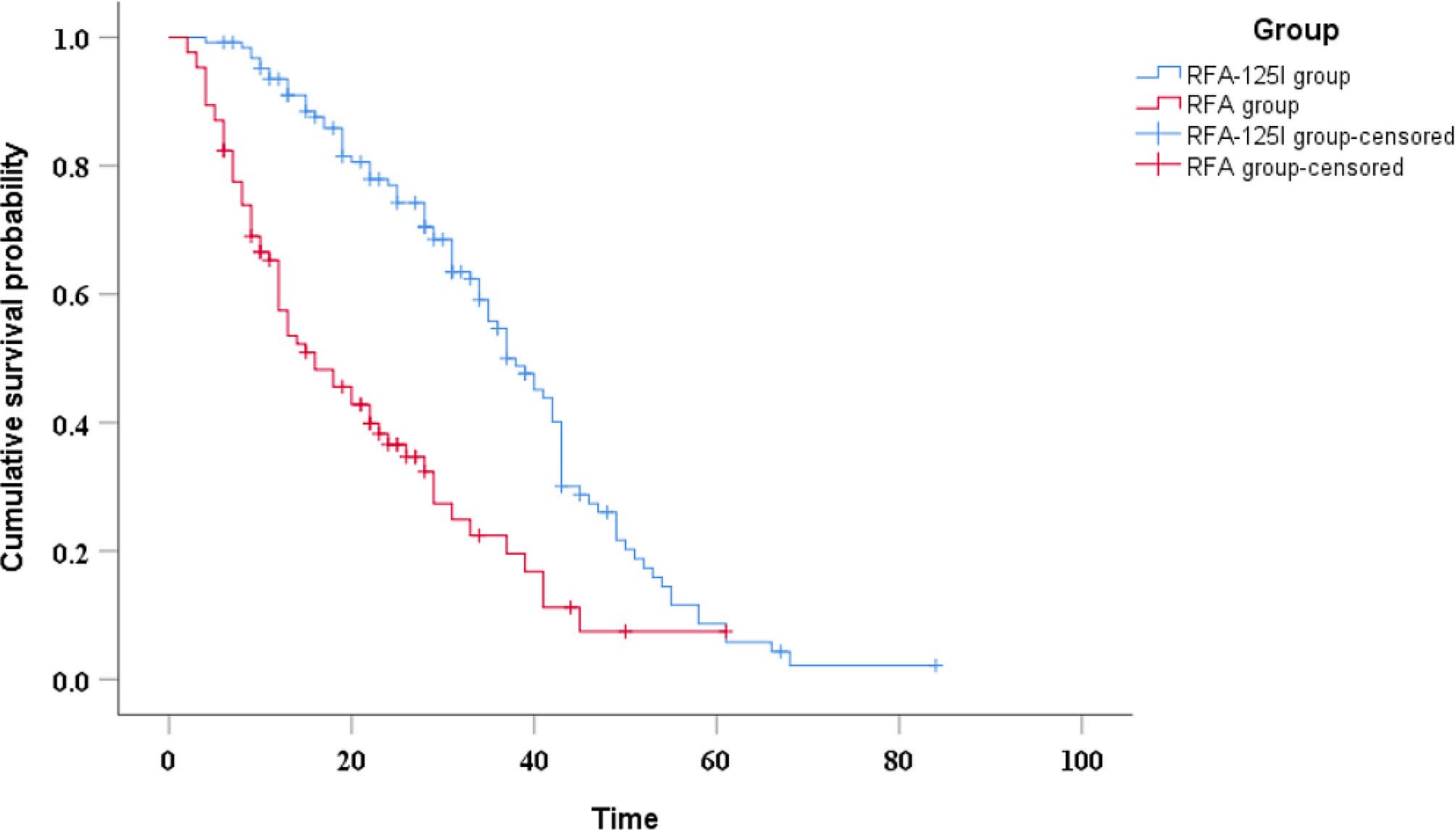
Kaplan–Meier curve of overall survival in HCC patients

The median OS was 16 months (95%CI, 10.0–22.0) in the RFA group and 37 months (95%CI, 32.1–41.9) in the RFA-^125^I group, with statistically significant differences between the two groups (*P* < 0.001) (Fig. 4). Univariable analysis indicated that platelet-to-lymphocyte ratio (PLR), neutrophil-to-lymphocyte ratio (NLR), tumor size, and treatment method were related to patients’ OS (Table 2). Including factors with significance < 0.1 into the multivariable analysis, the results showed that NLR and treatment method were independent prognostic factors affecting patients’ OS (*P* < 0.05) (Table 3).

**Table 2:** Univariate analysis of prognostic factors for overall survival and progression-free survival

Variables	OS		PFS	
	HR (95% CI)	*P* value	HR (95% CI)	*P* value
Gender				
Male	1		1	
Female	1.203 (0.755, 1.916)	0.436	0.946 (0.613, 1.459)	0.801
Age (years)	1.006 (0.991, 1.022)	0.433	1.009 (0.995, 1.024)	0.224
Hepatitis				
Hepatitis B	1		1	
Other	0.945 (0.618, 1.446)	0.794	1.038 (0.687, 1.570)	0.858
Child-Pugh score				
A	1			
B	0.849 (0.573, 1.256)	0.412	0.790 (0.540, 1.156)	0.225
TB (µmol/L)	1.009 (0.996, 1.022)	0.157	1.001 (0.988, 1.013)	0.923
Albumin (g/L)	1.001 (0.973, 1.030)	0.952	0.988 (0.960, 1.017)	0.424
PT (s)	0.967 (0.871, 1.074)	0.532	0.962 (0.878, 1.054)	0.402
AST (µmol/L)	1.004 (0.999, 1.008)	0.101	1.000 (0.996, 1.004)	0.952
ALT (µmol/L)	1.004 (0.999, 1.008)	0.101	1.000 (0.996, 1.004)	0.952
PLR	1.002 (1.000, 1.003)	0.049	1.002 (1.000, 1.003)	0.019
NLR	1.065 (1.016, 1.116)	0.008	1.072 (1.022, 1.124)	0.004
Tumor size	0.819 (0.649, 1.034)	0.093	1.099 (0.864, 1.399)	0.442
Tumor number				
1	1		1	
2–3	1.009 (0.692, 1.472)	0.963	0.991 (0.697, 1.409)	0.960
α-Fetoprotein level				
<400 ng/ml	1		1	
≥ 400 ng/mL	1.158 (0.826, 1.623)	0.394	0.919 (0.673, 1.257)	0.598
Treatment method				
RFA	1		1	
RFA-^125^I	0.383 (0.272,0.539)	0.000	0.602 (0.441, 0.823)	0.001

Note. OS: Overall survival; PFS: progression-free survival; HR: Hazard ratio; CI: Confidence interval; SD: Standard deviation; TB: Total bilirubin; PT: Prothrombin time; AST: Aspartate aminotransferase; ALT: Alanine aminotransferase; PLR: Platelet-to-lymphocyte ratio; NLR: Neutrophil-to-lymphocyte ratio; RFA: Radiofrequency ablation

**Table 3:** Multivariate analysis of prognostic factors for overall survival

Variables	HR (95% CI)	*P* value
PLR	1.000 (0.998, 1.002)	0.761
NLR	1.070 (1.007, 1.136)	0.028
Tumor size	0.824 (0.643, 1.055)	0.124
Treatment method		
RFA	1	
RFA-^125^I	0.395 (0.280, 0.557)	0.000

Note. HR: Hazard ratio; CI: Confidence interval; PLR: Platelet-to-lymphocyte ratio; NLR: Neutrophil-to-lymphocyte ratio; RFA: Radiofrequency ablation

### PFS

**Fig. 5:**
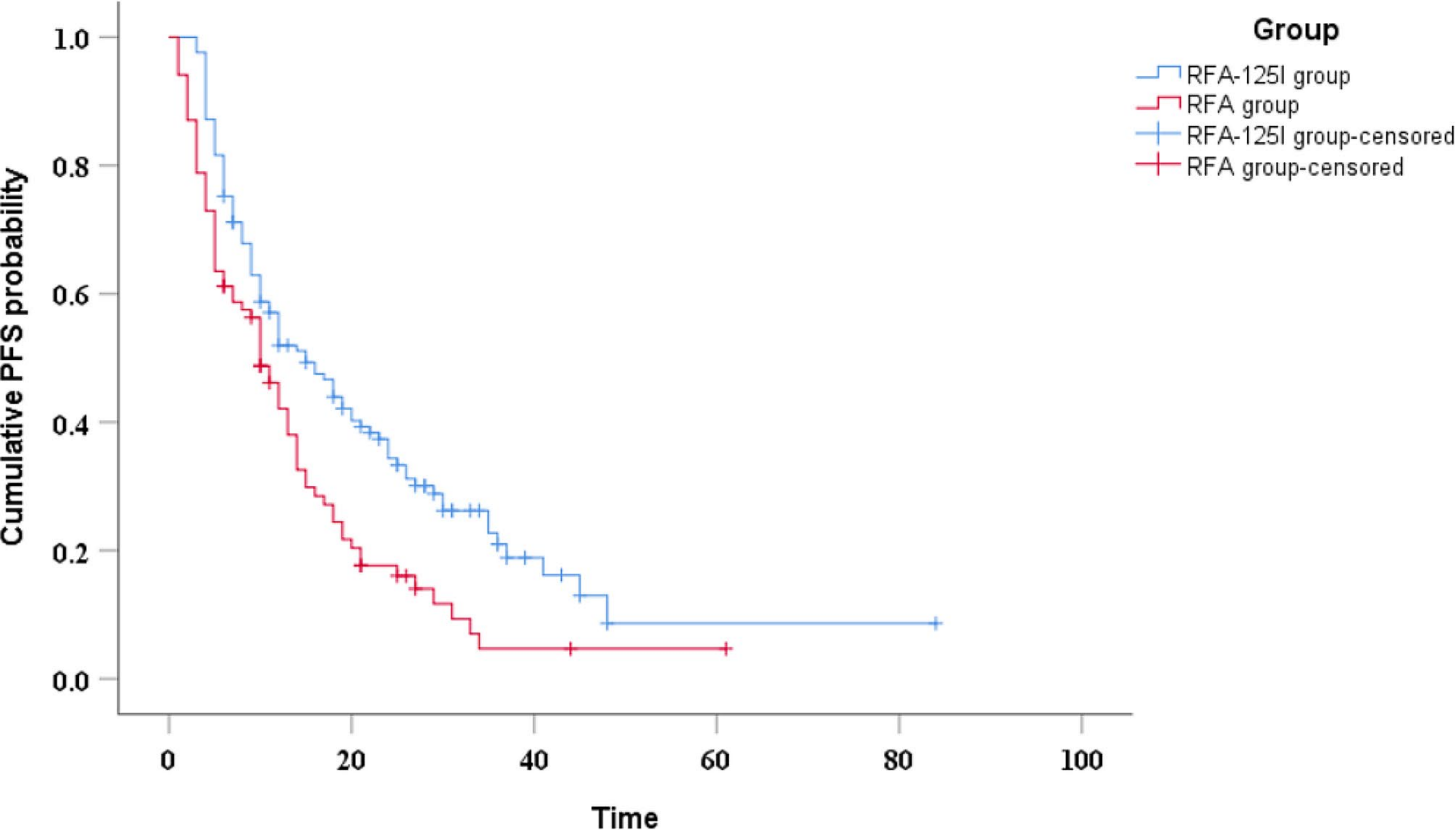
Kaplan–Meier curve of progression-free survival in HCC patients

The median PFS was 10 months (95%CI, 7.7–12.3) in the RFA group and 15 months (95%CI, 10.2–19.8) in the RFA-^125^I group (*P* = 0.001) (Fig. 5). Univariable analysis indicated that PLR, NLR and treatment method were related to patients’ PFS (Table 2). Including factors with significance < 0.1 into the multivariable analysis, the results showed that treatment method was independent prognostic factors affecting patients’ PFS (*P* < 0.05) (Table 4).

**Table 4:** Multivariate analysis of prognostic factors for progression-free survival

Variables	HR (95% CI)	*P* value
PLR	1.001 (0.999, 1.003)	0.404
NLR	1.057 (1.000, 1.119)	0.051
Treatment method		
RFA	1	
RFA-^125^I	0.626 (0.459, 0.855)	0.003

Note. HR: Hazard ratio; CI: Confidence interval; PLR: Platelet-to-lymphocyte ratio; NLR: Neutrophil-to-lymphocyte ratio; RFA: Radiofrequency ablation

## Discussion

In recent years, the application of I^125^ seeds implantation in the treatment of some malignant solid tumors such as HCC has expanded the indication of brachytherapy, and has been proved to be a good therapeutic effect. Our results indicated that the PFS of patients in the RFA-I^125^ group was significantly better than that of patients in the RFA group. Chen et al. showed in a randomized controlled study that for small HCCs, RFA-I^125^ can significantly control tumor recurrence compared with RFA alone [18]. Synergies between radiotherapy and thermal ablation have been reported to have “reciprocal zones of efficacy” [19]. The potential mechanisms of RFA and I^125^ seeds combination therapy for recurrent HCC are as follows: [1] Hyperthermia, increased vasodilation and vascular permeability in the peripheral area increase oxygenation in the area, further improving the efficacy of radiotherapy; [2] tumors with a low mutation burden and fewer neoantigens are generally less immunogenic, so they have little response to immunotherapy. However, the combination of RFA and I^125^ can not only reduce the tumor load of the body, but also promote the release of neoantigens, and effectively promote the lymphocyte infiltration of tumor tissues, improve the inhibition of tumor immune microenvironment, and ultimately promote the immune response [18].

It is well known that the control of intrahepatic lesions is essential for the survival of patients. A study showed that I^125^ brachytherapy can prolong PFS in patients with locoregional recurrence and/or residual HCC after RFA [20]. At the same time, the median OS was extended by 5 months. The results of this study also showed that patients in the RFA-I^125^ group had significantly prolonged PFS, and therefore, these patients’ OS was also better than that in the RFA group. Hence, It is beneficial for the long-term survival of HCC patients to control all target lesions as much as possible.

Chen et al. used MWA combined with I^125^ seeds to treat unresectable HCC in high-risk locations [17]. Both MWA and RFA belong to thermal ablation. Compared with RFA, MWA has the advantages of larger ablation range, shorter operation time, higher temperature of delivery to the target lesion, and less influence of heat sink effect. However, as the necrotic area expands, the risk of potential complications increases due to collateral damage to adjacent non-target organs [21, 22]. Compared with MWA, RFA is characterized by a slower heating rate, thus reducing the risk of thermal damage in the tissues surrounding the tumor ablation. Hence, in this study, we used RFA to treat recurrent HCC with the aim of further reducing the incidence of complications in patients.

Efficacy and safety are equally important for the treatment of HCC in high-risk locations. In addition to the correct selection of thermal ablation therapy, the choice of ablation/seeds implantation puncture path is also particularly important, and the choice of imaging method plays an important role in the formulation of the appropriate puncture path. Lin et al. reported the use of MRI-guided RFA/^125^I seeds therapy for HCC near large vessels, but the operation time was long and magnetic compatible puncture needles and RFA needles were required, which significantly limited its clinical application [16]. In addition, Chen et al. applied CT-guided MWA and ^125^I seeds implantation [17]. However, due to the inconsistent respiratory movements of patients under CT, the puncture angle and path need to be adjusted repeatedly. Therefore, this study applied RFA/I^125^ seeds implantation under the guidance of ultrasound and CT, and achieved good therapeutic effect and safety.

There are some limitations in this study. This study is a retrospective analysis, and the results may be subject to selection bias. Therefore, a multicenter prospective study is necessary to verify our results.

## Conclusion

For HCC that recurred after hepatectomy, RFA-I^125^ treatment was associated with better tumor control and long-term survival compared to RFA treatment. Meanwhile, ultrasound and CT guided puncture is safe and reliable.

## Data Availability

All data that support the findings of this study are collected objectively and are available from the corresponding author on reasonable request.

## References

[1] Sung H, Ferlay J, Siegel RL (2021). Global cancer statistics 2020: GLOBOCAN estimates of incidence and mortality worldwide for 36 cancers in 185 countries. CA Cancer J Clin.

[2] McGlynn KA, Petrick JL, El-Serag HB (2021). Epidemiology of hepatocellular carcinoma. Hepatology.

[3] Maria R, Alejandro F, Jordi R (2021). BCLC strategy for prognosis prediction and treatment recommendation: the 2022 update. J Hepatol.

[4] Zhang H, Liu F, Wen N (2022). Patterns, timing, and predictors of recurrence after laparoscopic liver resection for hepatocellular carcinoma: results from a high-volume HPB center. Surg Endosc.

[5] Yan WT, Li C, Yao LQ (2023). Predictors and long-term prognosis of early and late recurrence for patients undergoing hepatic resection of hepatocellular carcinoma: a large-scale multicenter study. Hepatobiliary Surg Nutr.

[6] Jeon MY, Kim HS, Lim TS (2019). Refractoriness to transarterial chemoembolization in patients with recurrent hepatocellular carcinoma after curative resection. PLoS ONE.

[7] Koh PS, Chan AC, Cheung TT (2016). Efficacy of radiofrequency ablation compared with transarterial chemoembolization for the treatment of recurrent hepatocellular carcinoma: a comparative survival analysis. HPB (Oxford).

[8] Lee DH, Lee MW, Kim PN (2021). Outcome of No-Touch Radiofrequency ablation for small Hepatocellular Carcinoma: a Multicenter Clinical Trial. Radiology.

[9] Wang Q, Tang M, Zhang S (2021). Comparison of radiofrequency ablation and surgical resection for hepatocellular carcinoma conforming to the Milan criteria: a meta-analysis. ANZ J Surg.

[10] Bai XM, Cui M, Yang W et al. The 10-year Survival Analysis of Radiofrequency Ablation for Solitary Hepatocellular Carcinoma 5 cm or Smaller: Primary versus Recurrent HCC. Radiology. 2021;300:458 – 69. 10.1148/radiol.2021200153 34003058

[11] Justin PM, Shota Y, Steven SR (2010). Percutaneous ablation of hepatocellular carcinoma: current status. J Vasc Interv Radiol.

[12] Künzli BM, Abitabile P, Maurer CA (2011). Radiofrequency ablation of liver tumors: actual limitations and potential solutions in the future. World J Hepatol.

[13] Ren YQ, Dong XJ, Chen L (2021). Combined Ultrasound and CT-Guided Iodine-125 seeds implantation for Treatment of Residual Hepatocellular Carcinoma Located at Complex sites after transcatheter arterial chemoembolization. Front Oncol.

[14] Zhang Y, Fan Y, Dong Z (2019). Iodine-125 implantation with transjugular intrahepatic portosystemic shunt for main portal vein tumor thrombus. World J Gastrointest Oncol.

[15] Chen L, Ying XH, Zhang DK (2020). Iodine-125 Brachytherapy can prolong progression-free survival of patients with Locoregional recurrence and/or residual Hepatocellular Carcinoma after Radiofrequency ablation. Cancer Biother Radiopharm.

[16] Lin Z, Chen J, Deng XF (2012). Treatment of hepatocellular carcinoma adjacent to large blood vessels using 1.5T. Eur J Radiol.

[17] Chen ZX, Fu XB, Qiu ZK (2023). CT-guided (125)I brachytherapy for hepatocellular carcinoma in high-risk locations after transarterial chemoembolization combined with microwave ablation: a propensity score-matched study. Radiol Oncol.

[18] Chen KY, Chen GH, Wang HN (2014). Increased survival in hepatocellular carcinoma with iodine-125 implantation plus radiofrequency ablation: a prospective randomized controlled trial. J Hepatol.

[19] Grieco CA, Simon CJ, Mayo-Smith WW (2006). Percutaneous image-guided thermal ablation and radiation therapy: outcomes of combined treatment for 41 patients with inoperable stage I/II nonsmall-cell lung cancer. J Vasc Interv Radiol.

[20] Chen L, Ying X, Zhang D (2021). Iodine-125 Brachytherapy can prolong progression-free survival of patients with Locoregional recurrence and/or residual Hepatocellular Carcinoma after Radiofrequency ablation. Cancer Biother Radiopharm.

[21] Izzo F, Granata V, Grassi R (2019). Radiofrequency ablation and microwave ablation in liver tumors: an update. Oncologist.

[22] Lucchina N, Tsetis D, Ierardi AM (2016). Current role of microwave ablation in the treatment of small hepatocellular carcinomas. Ann Gastroenterol.

